# New Books

**Published:** 2005-10

**Biodefense: Principles and Pathogens**

Michael S. Bronze, Ronald A. Greenfield, eds.

Norwich, UK:Horizon Scientific Press, 2005. 838 pp. ISBN: 1-904933-12-2, $380

**Chemoinformatics in Drug Discovery**

Tudor I. Oprea, Raimund Mannhold, Hugo Kubinyi, Gerd Folkers, eds.

Hoboken, NJ:John Wiley & Sons, Inc., 2005. 515 pp. ISBN: 3-527-30753-2, $185

**Climate Crash: Abrupt Climate Change and What It Means for Our Future**

John D. Cox

Washington, DC:National Academies Press, 2005. 224 pp. ISBN: 0-309-09312-0, $27.95

**Decision Making for the Environment: Social and Behavioral Science Research Priorities**

Garry D. Brewer, Paul C. Stern, eds.

Washington, DC:National Academies Press, 2005. 296 pp. ISBN: 0-309-09540-9, $45

**Encyclopedia of Genetics, Genomics, Proteomics and Bioinformatics**

Michael J. Dunn, Lynn B. Jorde, Peter F.R. Little, Shankar Subramaniam, eds.

Hoboken, NJ:John Wiley & Sons, Inc., 2005. 3,700 pp. ISBN: 0-471-84974-6, $1,660

**Environmental Economics for Tree Huggers and Other Skeptics**

William K. Jaeger

Washington, DC:Island Press, 2005. 240 pp. ISBN: 1-55963-668-8, $22.50

**EU Environmental Law: Challenges, Change and Decision-Making**

Maria Lee

Oxford, UK:Hart Publishing, 2005. 270 pp. ISBN: 1-84113-410-4, $50

**Fundamentals of Ecogenetics**

Lucio G. Costa, David L. Eaton, eds.

Hoboken, NJ:John Wiley & Sons, Inc., 2005. 516 pp. ISBN: 0-471-46781-2, $110

**Global Public Health Communication: Challenges, Perspectives and Strategies**

Muhiuddin Haide

Sudbury, MA:Jones and Bartlett Publishers, 2005. 450 pp. ISBN: 0-7637-4776-9, $68.95

**International Public Health: Diseases, Programs, Systems, and Policies**

Michael Merson, Robert Black, Anne Mills

Sudbury, MA:Jones and Bartlett Publishers, 2005. 775 pp. ISBN: 0-7637-2874-8, $87.95

**Nutrigenomics: Concepts and Technologies**

James Kaput, Raymond L. Rodriguez, eds.

Hoboken, NJ:John Wiley & Sons, Inc., 2005. 320 pp. ISBN: 0-471-68319-1, $79.95

**Proteomics for Biological Discovery**

Timothy D. Veenstra, John R. Yates

Hoboken, NJ:John Wiley & Sons, Inc., 2005. 320 pp. ISBN: 0-471-16005-9, $59.95

**Scarcity and Growth Revisited : Natural Resources and the Environment in the New Millennium**

R. David Simpson, Michael A. Toman, Robert U. Ayres

Washington, DC:RFF Press, 2005. 320 pp. ISBN: 1-933115-10-6, $70

**The Politics of the Global Oil Industry**

Toyin Falola, Ann Genova

Westport, CT:Praeger, 2005. 280 pp. ISBN: 0-275-98400-1, $44.95

**Toxicogenomic Technologies and Risk Assessment of Environmental Carcinogens: A Workshop Summary**

Committee on How Toxicogenomics Could Inform Critical Issues in Carcinogenic Risk Assessment of Environmental Chemicals, Committee on Emerging Issues and Data on Environmental Contaminants, National Research Council

Washington, DC:National Academies Press, 2005. 55 pp. ISBN: 0-309-09700-2, $12

